# Supplementary material for: A New Gal in Town: A Systematic Review of the Role of Galanin and Its Receptors in Experimental Pain
Source: Cells. 2022 Mar 1;11(5):839. doi: 10.3390/cells11050839 (PMC8909084; doi:10.3390/cells11050839)
Supplement: Supplementary file 1 [file cells-11-00839-s001.zip › cells-1604398-supplementary.pdf]

Table S1. Summary of the methodological quality of the included studies, assessed using the ARRIVE guidelines for the reporting of in vivo experiments. Summarised criteria: 1 – Study design; 2 – Sample size; 3 – Inclusion and exclusion criteria; 4 – Randomisation; 5 – Blinding; 6 – Outcome measures; 7 – Statistical methods; 8 – Experimental animals; 9 – Experimental procedures; 10 – Results; 11 – Abstract; 12 – Background; 13 – Objectives; 14 – Ethical statement; 15 – Housing and husbandry; 16 – Animal care and monitoring; 17 – Interpretation/scientific implications; 18 – Generalisability; 19 – Protocol registration; 20 – Data access; 21 – Declaration of interests (Red: absent, yellow: incomplete, green: complete).

	1	2	3	4	5	6	7	8	9	10	11	12	13	14	15	16	17	18	20	21
Post et al., 1988 (1)	Green	Yellow	Red	Yellow	Green	Yellow	Yellow	Green	Green	Yellow	Red	Yellow	Green	Red	Red	Red	Yellow	Yellow	Red	Red
Wiesenfeld-Hallin et al., 1990 (2)	Yellow	Red	Green	Red	Red	Yellow	Red	Green	Green	Yellow	Yellow	Yellow	Green	Red	Red	Red	Yellow	Green	Red	Yellow
Xu et al., 1991 (3)	Red	Red	Red	Red	Red	Green	Yellow	Green	Green	Green	Yellow	Green	Green	Red	Red	Red	Yellow	Yellow	Red	Red
Kuraishi et al., 1991a (4)	Red	Red	Red	Red	Red	Green	Yellow	Green	Red	Green	Yellow	Yellow	Red	Yellow	Green	Red	Yellow	Yellow	Red	Red
Kuraishi et al., 1991b (5)	Red	Red	Red	Red	Red	Green	Red	Green	Green	Green	Yellow	Yellow	Green	Red	Red	Red	Yellow	Yellow	Red	Red
Wiesenfeld-Hallin et al., 1993 (6)	Red	Red	Red	Red	Green	Yellow	Red	Green	Green	Green	Red	Yellow	Green	Yellow	Red	Red	Yellow	Green	Red	Yellow
Reimann et al., 1994 (7)	Red	Red	Red	Red	Red	Green	Green	Green	Green	Yellow	Green	Green	Green	Red	Red	Red	Green	Green	Red	Red
Przewlocka et al., 1995 (8)	Red	Red	Red	Red	Red	Green	Yellow	Green	Green	Yellow	Yellow	Yellow	Green	Red	Green	Yellow	Green	Yellow	Red	Yellow
Xu et al., 1997a (9)	Green	Green	Red	Red	Green	Green	Green	Green	Green	Green	Green	Green	Green	Green	Red	Yellow	Green	Green	Red	Yellow
Xu et al., 1997b (10)	Red	Red	Red	Red	Red	Green	Green	Green	Green	Yellow	Yellow	Green	Green	Green	Red	Green	Green	Yellow	Red	Yellow
Wang et al., 1999 (11)	Green	Yellow	Green	Red	Red	Green	Yellow	Green	Green	Green	Yellow	Yellow	Green	Green	Red	Red	Red	Yellow	Red	Yellow
Rezaei et al., 2000 (12)	Red	Red	Green	Red	Red	Red	Yellow	Green	Green	Yellow	Yellow	Yellow	Green	Red	Red	Green	Yellow	Yellow	Red	Yellow
Blakeman et al., 2001 (13)	Red	Red	Red	Red	Green	Green	Yellow	Yellow	Yellow	Yellow	Yellow	Green	Green	Green	Yellow	Green	Green	Yellow	Red	Yellow
Grass et al., 2003a (14)	Green	Yellow	Green	Red	Red	Green	Green	Green	Green	Yellow	Yellow	Green	Green	Green	Yellow	Red	Green	Yellow	Red	Yellow
Grass et al., 2003b (15)	Red	Red	Green	Red	Red	Green	Green	Green	Green	Yellow	Yellow	Green	Green	Yellow	Yellow	Green	Green	Yellow	Red	Yellow
Ackermann et al., 2003 (16)	Red	Red	Red	Red	Red	Red	Yellow	Green	Green	Yellow	Yellow	Yellow	Green	Green	Yellow	Red	Yellow	Yellow	Red	Yellow
Hua et al., 2004 (17)	Green	Yellow	Red	Red	Red	Green	Green	Green	Green	Yellow	Green	Yellow	Red	Green	Green	Yellow	Green	Green	Red	Yellow
Sun & Yu, 2005 (18)	Red	Red	Green	Red	Red	Green	Yellow	Green	Green	Yellow	Yellow	Yellow	Green	Red	Yellow	Yellow	Yellow	Yellow	Red	Yellow
Wu & Yu, 2006 (19)	Green	Yellow	Red	Red	Red	Green	Yellow	Green	Green	Green	Yellow	Yellow	Green	Yellow	Green	Green	Green	Yellow	Red	Yellow
Sun et al., 2007 (20)	Green	Yellow	Red	Red	Red	Green	Yellow	Green	Green	Green	Green	Green	Green	Yellow	Yellow	Green	Green	Yellow	Red	Yellow
Zubrzycka & Janecka, 2007 (21)	Green	Yellow	Green	Red	Red	Green	Yellow	Green	Green	Green	Green	Yellow	Green	Red	Red	Green	Green	Yellow	Red	Yellow

Zubrzycka & Janecka, 2008 (22)																				
Allier et al., 2008 (23)																				
Hobson et al., 2009 (24)																				
Jin et al. 2010 (25)																				
Fu et al., 2011 (26)																				
Shi et al., 2011 (27)																				
Yue et al., 2011 (28)																				
Lemons & Wiley, 2011 (29)																				
Li et al., 2012 (30)																				
Xu et al, 2012 (31)																				
Kong & Yu, 2013 (32)																				
Zhang et al., 2015 (33)																				
Pulse et al., 2016 (34)																				
Fu et al., 2016 (35)																				
Taylor et al., 2020 (36)																				
Wiesenfeld-Hallin et al., 1989 (37)																				
Satoh et al, 1992 (38)																				
Wiesenfeld-Hallin et al., 1992 (39)																				
Lundeberg et al., 1993 (40)																				
Zhang et al., 1993 (41)																				
Verge et al., 1993 (42)																				
Hope et al., 1994 (43)																				
Nahin et al., 1994 (44)																				
Ji et al., 1994 (45)																				
Luo & Wiesenfeld-Hallin, 1995 (46)																				
Xu et al., 1996 (47)																				
Barajon et al., 1996 (48)																				
Munglani et al., 1996 (49)																				

Rydh-Rinder et al., 1996 (50)																				
Carlton et al., 1996 (51)																				
Ji et al., 1996 (52)																				
Corness et al., 1996 (53)																				
Calzá et al., 1997 (54)																				
Sten Chi et al., 1997 (55)																				
Ma & Bisby, 1997 (56)																				
Long et al., 1998																				
Sten Chi et al., 1998																				
Fukuoka et al., 1998																				
Colvin et al., 1998																				
Ma & Bisby, 1999a																				
Long-Chuan et al., 1999																				
Ma & Bisby, 1999b																				
Sten Chi et al., 1999																				
Hao et al., 1999 (57)																				
Calzá et al., 2000 (58)																				
Honore et al., 2000 (59)																				
Wang et al., 2000 (60)																				
Liu et al., 2000 (61)																				
Xu et al., 2000 (62)																				
Zhang et al., 2000a (63)																				
Zhang et al., 2000b (64)																				
Bradley et al., 2001 (65)																				
Hu & McLachlan, 2001 (66)																				
Liu et al., 2001 (67)																				

Macdonald et al., 2001 (68)																				
Kerr et al., 2001 (69)																				
Bird et al., 2002 (70)																				
Flatters et al., 2002 (71)																				
Bao et al., 2002 (72)																				
Sun et al., 2003 (73)																				
Blakeman et al., 2003 (74)																				
Wang et al., 2003 (75)																				
Flatters et al., 2003 (76)																				
Hofmann et al., 2003 (77)																				
Jimenez-Andrade et al., 2004 (78)																				
Sun et al., 2004 (79)																				
Imbe et al., 2004 (80)																				
Hygge-Blakeman et al., 2004 (81)																				
Peters et al., 2005 (82)																				
Xiong et al., 2005 (83)																				
Jimenez-Andrade et al., 2005 (84)																				
Malkmus et al., 2005 (85)																				
Garry et al., 2005 (86)																				
Lu et al., 2005 (87)																				
Holmberg et al., 2005 (88)																				
Jimenez-Andrade et al., 2006 (89)																				
Shi et al., 2006 (90)																				
Brumovsky et al., 2006 (91)																				
Wilson-Gerwing & Verge, 2006 (92)																				
Wallace et al., 2007 (93)																				
Wang et al., 2007 (94)																				
Gu et al., 2007 (95)																				

Nishii et al., 2007 (96)																				
Hulse et al., 2008 (97)																				
Coronel et al., 2008 (98)																				
Jung et al., 2009 (99)																				
Coronel et al., 2009 (100)																				
An et al., 2010 (101)																				
Pope et al., 2010 (102)																				
Zhang et al., 2011 (103)																				
Hulse et al., 2011 (104)																				
Hulse et al., 2012 (105)																				
Yang et al., 2012 (106)																				
Xu et al., 2012 (107)																				
Amorim et al., 2014 (108)																				
Su et al., 2015 (109)																				
Yang et al., 2015 (110)																				
Metcalf et al., 2015 (111)																				
Reinhold et al., 2015 (112)																				
Duan et al., 2015 (113)																				
Boateng et al., 2015 (114)																				
Wu et al., 2016 (115)																				
Xu et al., 2016 (116)																				
Zhang et al., 2016 (117)																				
Boltz et al., 2016 (118)																				
Zhang et al., 2017 (119)																				
Holmes et al., 2017 (120)																				
Coronel et al., 2017 (121)																				
(122)																				
Li et al., 2017 (123)																				

Chen et al., 2018 (124)																				
Fernandez-Zafra et al., 2019 (125)																				
Zhang et al., 2019a (126)																				
Zhang et al., 2019b (127)																				
Lyu et al., 2020 (128)																				
Liu et al., 2020 (129)																				
Li et al., 2021 (130)																				
Dong et al., 2021 (131)																				

Table S2. Summary of the literature search on the role of galanin and its receptors in the processing of nociceptive information.

Article	Results - Galanin	Results - Galanin Receptors	Experimental subjects
Post et al., 1988 (1)	Intrathecal administration of galanin - antinociceptive effect (thermal and mechanical sensitivity)	-	Male NRMI mice
Wiesenfeld-Hallin et al., 1990 (2)	Intrathecal administration of galanin - no effect (flexor reflex). Galanin potentiates the analgesic effect of morphine.	-	Male Sprague–Dawley rats
Xu et al., 1991 (3)	Intrathecal administration of galanin - antinociceptive effect (flexor reflex)	-	Male Sprague–Dawley rats
Kuraishi et al., 1991a (4)	Intrathecal administration of galanin - antinociceptive effect (thermal and mechanical sensitivity). Galanin present in the dorsal horn is involved in the transmission of mechanical, but not thermal nociceptive information.	-	Male Sprague-Dawley rats
Kuraishi et al., 1991b (5)	Intrathecal administration of galanin - antinociceptive effect (thermal sensitivity). Substance P is involved in galanin-induced antinociception.	-	Male Sprague–Dawley rats
Wiesenfeld-Hallin et al., 1993 (6)	Intrathecal administration of galanin - antinociceptive effect (thermal and mechanical sensitivity)	-	Male Sprague-Dawley rats
Reimann et al., 1994 (7)	Role of endogenous galanin in nociceptive processing – antinociceptive effect. Galanin is involved in the spinal effects of morphine.	-	Male Sprague-Dawley rats
Przewlocka et al., 1995 (8)	Intracerebroventricular administration of galanin - no effect (thermal and mechanical sensitivity). Galanin potentiates the analgesic effect of morphine.	-	Male Wistar rats
Xu et al., 1997a (9)	Increased galanin levels in sensory neurons after RTX	-	Male Sprague-Dawley rats
Xu et al., 1997b (10)	Intrathecal galanin - antinociceptive effect (reduced spinal flexor reflex hyperexcitability)	-	Female Sprague-Dawley rats
Wang et al., 1999 (11)	Intra-periaqueductal grey injection - antinociceptive effect (thermal and mechanical sensitivity). Interaction between galanin and opioids.	-	Male Sprague–Dawley rats
Rezaei et al., 2000 (12)	Intrathecal administration of galanin - antinociceptive effect (flexor reflex)	Inactivation of GalR1 - blockade of the inhibitory effect of galanin	Female Sprague-Dawley rats
Blakeman et al., 2001 (13)	Overexpression of galanin - antinociceptive effect (thermal and mechanical sensitivity)	-	Both male and female Gal-OE mice
Grass et al., 2003a (14)	Overexpression of galanin – antinociceptive effect (reduced facilitation of the nociceptive flexor reflex)	-	Both male and female Gal-OE mice

Grass et al., 2003b (15)	Intrathecal administration of galanin – antinociceptive effect (flexor reflex)	Galanin antinociceptive effect is probably mediated by receptors other than GalR1	Male GalR1-KO mice
Ackermann et al., 2003 (16)	Increased galanin – antinociceptive effect (mechanical sensitivity)	-	Male Sprague-Dawley rats
Hua et al., 2004 (17)	Intrathecal administration of Galanin - antinociceptive effect (formalin-induced nociception).<br>Galanin potentiates intrathecal morphine and AP-5-induced antinociception.	The antinociceptive effect of intrathecal galanin is mediated by activation of spinal GalR1, but not GalR2 receptors	Male Sprague-Dawley rats
Sun & Yu, 2005 (18)	Injection of galanin to the arcuate nucleus of the hypothalamus - antinociceptive effect (thermal and mechanical sensitivity)	-	Male Wistar Han rats
Wu & Yu, 2006 (19)	Intracerebroventricular administration of galanin - antinociceptive effect (thermal and mechanical sensitivity)	-	Male Wistar rats
Sun et al., 2007 (20)	Injection of galanin to the arcuate nucleus of the hypothalamus - antinociceptive (thermal and mechanical sensitivity).<br>Galaninergic fibres directly innervate β-endorphinergic neurons in this area.	-	Male Wistar rats
Zubrzycka & Janecka, 2007 (21)	Intracerebroventricular administration of galanin – antinociceptive effect (trigemino-hypoglossal reflex)	-	Male Long-Evans rats
Zubrzycka & Janecka, 2008 (22)	Intracerebroventricular administration of galanin - antinociceptive (trigemino-hypoglossal reflex).<br>Galanin exerts its antinociceptive effects in the orofacial area through the μ-opioid receptor.	-	Male Long-Evans rats
Allier et al., 2008 (23)		(1) GalR1 receptors are located predominantly post-synaptically whereas GalR2 receptors may be localized both pre- and post-synaptically, (2) activation of either GalR1 or GalR2 can have both antinociceptive and pronociceptive effects	Both male and female Sprague-Dawley rats
Hobson et al., 2009 (24)		The absence of GalR2 expression induces the loss of a subset of sensory neurons	Male GalR2-MUT mice
Jin et al. 2010 (25)	Injection of galanin to the central nucleus of the amygdala - antinociceptive effect (thermal and mechanical sensitivity).<br>Both mu- and delta-opioid receptors are involved in galanin-induced antinociception.		Male Wistar rats
Fu et al., 2011 (26)	Intracerebroventricular administration of galanin - antinociceptive effect (thermal and mechanical sensitivity)	GalR1 as an antinociceptive target in the brain	Male Sprague-Dawley rats
Shi et al., 2011 (27)	Intracerebroventricular administration of galanin - antinociceptive effect (thermal and mechanical sensitivity).		Male Sprague-Dawley rats

	PKC is involved in galanin-induced antinociception		
Yue et al., 2011 (28)	Galanin at lower concentrations enhances the spontaneous release of L-glutamate onto substantia nigra neurons from nerve terminals by activating GalR2/R3, whereas galanin at higher concentrations also produces a membrane hyperpolarization by activating GalR1		Male Sprague-Dawley rats
Lemons & Wiley, 2011 (29)		Selective destruction of GalR1-expressing superficial dorsal horn neurons produces heat hypoalgesia	Female Long Evans hooded rats
Li et al., 2012 (30)	Injection of galanin to the central nucleus of the amygdala – antinociceptive effect (thermal and mechanical sensitivity)	GalR1 as an antinociceptive target in the central nucleus of the amygdala	Male Sprague-Dawley rats
Xu et al, 2012 (31)	Injection of galanin to the nucleus accumbens - antinociceptive effect (thermal and mechanical sensitivity)		Male Sprague-Dawley rats
Kong & Yu, 2013 (32)	Intra-periaqueductal grey injection antinociceptive effect (thermal and mechanical sensitivity)	Activation of GalR1 induces antinociception in rats with morphine tolerance.	Male Sprague-Dawley rats
Zhang et al., 2015 (33)	Intra-periaqueductal grey injection - antinociceptive (thermal and mechanical).<br>CAMKII may be involved in galanin-induced antinociception.	GalR2 antagonist administration (M871) - pronociceptive	Male Sprague-Dawley rats
Pulse et al., 2016 (34)	Administration of galanin to the saphenous nerve truck – antinociceptive effect (inhibits axonal excitability)		Male Wistar rats
Fu et al., 2016 (35)	Intra-lateral habenula complex injection - antinociceptive effect (thermal and mechanical sensitivity)	GalR1 as an antinociceptive target in the lateral habenula	Male Sprague-Dawley rats
Taylor et al., 2020 (36)	Galanin in the lumbar splanchnic nerve (LSN) – antinociceptive effect (mechanical sensitivity)	GalR1 activation, but not GalR2/3 activation, suppresses mechanosensitivity - GalR1 mediates the suppressive effect of galanin.	Male and female C57BL/6J mice

Table S3. Summary of the literature search on the role of galanin and its receptors in different animal models of experimental pain. ACC - anterior cingulate cortex; ARC – arcuate nucleus of the hypothalamus; DRG – dorsal root ganglia; NAc – nucleus accumbens.

**Article**	**Results - Galanin**	**Results - Galanin Receptors**	**Animal model**	**Experimental subjects**
Wiesenfeld-Hallin et al., 1989 (37)	Galanin had a biphasic effect on the flexor reflex in rats with intact nerves, including facilitation, followed by depression, in a dose-dependent manner.		Sciatic nerve axotomy	Sprague-Dawley rats
Satoh et al, 1992 (38)	Antinociceptive role of galanin - intrathecal injections of antibodies against galanin inhibited carrageenan-induced hyperalgesia		Repeated cold stress (RCS)-induced hyperalgesia + Carragenan	Male Sprague–Dawley rats
Wiesenfeld-Hallin et al., 1992 (39)	Antinociceptive role of galanin - M-35 (galanin receptor antagonist) has a facilitatory effect on flexor reflex excitability, which was potentiated after nerve axotomy		Sciatic nerve axotomy	Female Sprague-Dawley rats
Lundeberg et al., 1993 (40)	Galanin inhibits carrageenan-induced mechanical hyperalgesia by inhibiting the release of oxytocin from nerve terminals in the spinal cord		Carragenan-induced inflammation	Male Sprague-Dawley rats
Zhang et al., 1993 (41)	Increase in galanin-positive cells after axotomy (DRG)		Sciatic nerve axotomy	Female monkeys
Verge et al., 1993 (42)	Antinociceptive role of galanin - M-35 (galanin receptor antagonist) enhances autotomy behaviour in rats after sciatic nerve section		Sciatic nerve axotomy	Male Sprague–Dawley rats
Hope et al., 1994 (43)	Increase in galanin-positive cells after flexion of inflamed ankles (SDH)		Freund's adjuvant (ankle)	Both male and female rats
Nahin et al., 1994 (44)	Increase in galanin mRNA levels after CCI (primary sensory neurons)		Chronic constriction injury (CCI) of the sciatic nerve	Male Sprague-Dawley rats
Ji et al., 1994 (45)	Upregulation of galanin in DRG neurons after axotomy - antinociceptive		Sciatic nerve axotomy	Male Sprague-Dawley rats
Luo & Wiesenfeld-Hallin, 1995 (46)	Intrathecal galanin - antinociceptive effect (blocked both components of reflex hyperexcitability following sciatic nerve section).		Sciatic nerve axotomy	Female Sprague-Dawley rats
Xu et al., 1996 (47)	Upregulation of Galanin in DRG neurons after axotomy – antinociceptive effect. DRG neurones are sensitive to galanin.	GalR1 receptors are involved in sensory processing	Carrageenan + sciatic nerve axotomy	Male Sprague–Dawley rats

Barajon et al., 1996 (48)	Increased expression of Galanin in DRG, decreased in the spinal cord and sciatic nerve		Cisplatin	Female Wistar rats
Munglani et al., 1996 (49)	Decrease in galanin-positive cells (primary afferent neurones and spinal cord) after sciatic nerve compression		Chronic constriction injury (CCI) of the sciatic nerve	Glaxo-bred, random hooded rats
Rydh-Rinder et al., 1996 (50)	Moderate increase in galanin-positive cells in DRG and spinal cord after axotomy		Sciatic nerve axotomy	Male guinea-pigs
Carlton et al., 1996 (51)	Increase in galanin-positive cells after peripheral nerve injury (dorsal horn)		Spinal nerve ligation	Male monkeys
Ji et al., 1996 (52)	Upregulation of galanin in DRG neurons after axotomy		Sciatic nerve axotomy	Male Sprague-Dawley rats
Corness et al., 1996 (53)	Upregulation of Galanin in DRG after axotomy. Leukaemia inhibitory factor (LIF) is important for the dramatic upregulation of GAL seen after axotomy		Sciatic nerve axotomy	WT and leukaemia inhibitory factor (LIF)-deficient mice
Calzá et al., 1997 (54)	As soon as signs of inflammation appear, there is a decrease in immunostaining for galanin in fibres in the superficial layers of the spinal cord. Peptide content then gradually increases, so that after 21 days a recovery can be observed. Galanin is also expressed in interneurons on the superficial dorsal horn, and galanin mRNA expression increased in small neurons in layers I and II during chronic inflammation 21 days after CFA injection		Adjuvant arthritis	Male Sprague-Dawley rats
Sten Chi et al., 1997 (55)		GalR1 and GalR2 are expressed in rat DRG neurons under normal circumstances. Both GalR2 and GalR1 mRNA levels decrease after axotomy. Inflammation increased both GalR2 mRNA levels and number of positive neurons, whereas GalR1 is downregulated.	Carragenan + Sciatic nerve axotomy	Male Sprague–Dawley rats

Ma & Bisby, 1997 (56)	Increase in Galanin-positive cells after sciatic nerve injury in the ipsilateral dorsal root ganglia and the superficial laminae of the dorsal horn and the gracile nuclei. In partial injury models, the percentages of galanin-immunoreactive DGR neurons were significantly higher than in complete nerve transection.		Complete transection, partial transection and chronic constriction injury of the sciatic nerve	Male Sprague-Dawley rats
Long et al., 1998	Increased expression of Galanin within IAN neuromas at 3 days after nerve section and ligation		Inferior alveolar nerve neuroma (IAN)	Both male and female ferrets
Sten Chi et al., 1998	Galanin was upregulated in DRG neurons after peripheral axotomy		Sciatic nerve axotomy	Female C57/BL6 mice
Fukuoka et al., 1998	The numbers of neurons expressing galanin in the L5 DRG increased 7 days after axotomy. However, L5 spinal nerve ligation had no effect on the number of galanin mRNA-expressing neurons in neighbouring intact L4 DRG.		Spinal nerve ligation (SPNL) + Sciatic nerve axotomy	Male Sprague-Dawley rats
Colvin et al., 1998	High intensity electrical stimulation of the injured nerve resulted in a further increase in immunoreactive-galanin release in the superficial dorsal horn, with no significant persistence of galanin after release.		Chronic constriction injury (CCI) of the sciatic nerve	Rats
Ma & Bisby, 1999a	Two weeks after CCI, galanin-immunoreactive axonal fibres were dramatically increased in the ipsilateral gracile nuclei		Chronic constriction injury (CCI) of the sciatic nerve	Male Sprague-Dawley rats
Long-Chuan et al., 1999	Intrathecal administration of galanin – antinociceptive effect (thermal and mechanical stimulation)		Chronic constriction injury (CCI) of the sciatic nerve	Male Sprague–Dawley rats
Ma & Bisby, 1999b	4 and 14 days after PSNL the percentages of galanin mRNA positive neurons were significantly increased in the ipsilateral DRG compared to the contralateral side		Partial sciatic nerve ligation (PSNL)	Male Sprague–Dawley rats
Sten Chi et al., 1999	Increase in the number of galanin-immunoreactive and galanin messenger RNA-positive neuron profiles in ipsilateral DRG in all types of nerve injury models		Photochemical lesion + chronic constriction injury of the sciatic nerve + axotomy	Male Sprague-Dawley rats

Hao et al., 1999 (57)	Intrathecal galanin - antinociceptive effect (alleviated mechanical- and cold-allodynia-like behaviours)		Photochemically induced sciatic nerve injury	Male Sprague–Dawley rats
Calzá et al., 2000 (58)	Upregulation of galanin mRNAs in DRGs in both the acute and remission stages of arthritis, specially at time-course points with high inflammation and severe joint destruction		Adjuvant arthritis	Male Sprague–Dawley rats
Honore et al., 2000 (59)	Increase in galanin-expressing neurons in DRG		Sciatic nerve ligation (SNL) and nerve transection + CFA injection	Male C3H/HeJ mice
Wang et al., 2000 (60)	Intra-PAG administration of galanin - antinociceptive effect in rats with mononeuropathy. PAG is one of the key sites of the interaction between galanin and opioids in rats with mononeuropathy		Chronic constriction injury (CCI) of the sciatic nerve	Male Sprague-Dawley rats
Liu et al., 2000 (61)	Intrathecal administration of galanin - antinociceptive. M35 (galanin receptor antagonist) had no agonistic effect in the spinal cord		Chronic constriction injury (CCI) of the sciatic nerve	Male Sprague-Dawley rats
Xu et al., 2000 (62)	Galanin inhibited the activity of WDR neurons in a dose-dependent fashion. The effect of galanin was more pronounced in sciatic nerve ligated rats than intact rats. Administration of the galantide (galanin antagonist) - increase in the WDR neuron discharge frequency.		Chronic constriction injury (CCI) of the sciatic nerve	Male Sprague–Dawley rats
Zhang et al., 2000a (63)	Galanin acts synergically with opioids to inhibit the nociceptive information transmission.		Sciatic nerve ligation	Male Sprague–Dawley rats
Zhang et al., 2000b (64)	Specific interaction between galanin and opioids in the spinal cord of neuropathic rats, related to the activation of μ-opioid receptors.		Chronic constriction injury (CCI) of the sciatic nerve	Male Sprague-Dawley rats
Bradley et al., 2001 (65)	Knockout of galanin - antinociceptive. Gal-KO mice are hypo-responsive in the formalin test; display a decrease of post-CS facilitation of spinal reflexes and a decrease in spinal hyperexcitability following peripheral inflammation. Endogenous galanin is necessary for the full expression of central sensitization, and as such, plays a critical role in the development of hyperalgesia following peripheral tissue injury.		Carragenan	Adult mice of both sexes
Hu & McLachlan, 2001 (66)	Intrathecal administration of galanin – antinociceptive effect (mechanical stimulation) Intrathecal injection of a galanin		Kaolin/Carragenan model	Male Wistar rats

	antagonist increased the response of the peripheral nerve fibre endings			
Liu et al., 2001 (67)	Upregulation of spinal galanin expression following damage to a peripheral nerve, with no direct effect on basal spinal excitability		Sciatic nerve axotomy	Adult mice of both sexes
Macdonald et al., 2001 (68)	Upregulation of galanin in DRG somata after axotomy. Galanin+ axons sprout within the DRGs containing neurones that project into damaged peripheral nerves, forming varicose rings around DRG somata		Spinal or sciatic nerve axotomy	Female Wistar rats
Kerr et al., 2001 (69)	Intrathecal infusion of low-dose galanin in normal rats – pronociceptive effect (mechanical and cold allodynia)	A low dose of galanin has a nociceptive role at the spinal cord level mediated by GalR2 receptors, whereas the antiallodynic effect of high-dose galanin on neuropathic pain is mediated by the GalR1 receptors. Thus, a selective GalR1 agonist may be used to treat neuropathic pain.	Chronic constriction injury (CCI) of the sciatic nerve	Male Sprague-Dawley rats
Bird et al., 2002 (70)	Increased expression of galanin in the central region of IAN neuromas at 3 days after nerve section and ligation	No changes in expression of GalR2 following PSNL	Partial sciatic nerve ligation (PSNL)	Male Wistar rats
Flatters et al., 2002 (71)	Spinal administration of galanin - antinociceptive effect (inhibition of up to 80% of responses to both natural and electrical stimuli)		Chronic constriction injury (CCI) of the sciatic nerve	Male Wistar rats
Bao et al., 2002 (72)	After axotomy, galanin was expressed in many small and some large neurons in the spinal cord. Increase in galanin-nerve fibres in laminae I–II, with a very limited expansion into lamina III, 14 days after sciatic nerve transection		Inferior alveolar nerve neuroma (IAN)	Both male and female ferrets
Sun et al., 2003 (73)	Intra-ARC administration of galanin - antinociceptive effect. Both exogenous and endogenous galanin plays an antinociceptive role in the ARC		Carrageenan-induced inflammation	Male Wistar rats
Blakeman et al., 2003 (74)		GalR1 Knockout - pronociceptive (mechanical and thermal). Galanin produces antinociception in rodents through activation of GalR1	Photochemically induced sciatic nerve injury + sciatic nerve crush	Galr1−/−, wild type (Galr1+/+) and Galr1+/−
Wang et al., 2003 (75)	In SNL rats, upregulation of galanin-positive neurons in ipsilateral L5 DRG neurons and in the dorsal horn laminae I–III		Spinal nerve ligation (SNL)	Male Sprague-Dawley rats
Flatters et al., 2003 (76)	Galanin produced inhibitory effects in a larger proportion of the nociceptive fibres and spinal neurones examined and a		Spinal nerve ligation (SNL)	Male Sprague–Dawley rats

	more variable facilitatory effect in the remaining fibres and neurones			
Hofmann et al., 2003 (77)	L5 DRG mRNA expression of galanin was increased from day 2 to day 12 in tibial nerve injury rats		Tibial nerve injury	Male Wistar rats
Jimenez-Andrade et al., 2004 (78)	Intraplantar administration of galanin at low doses - pronociceptive effect (increases CAP-evoked nociceptive behaviours). Galanin does not modify activity of nociceptors but markedly enhances CAP-induced excitation of these fibres.	The pronociceptive effect of galanin at low doses is attributed to activation of peripheral GalR2. GalR2 are highly expressed in L5 dorsal root ganglion (DRG) cells	Capsaicin (CAP)	Male Sprague-Dawley rats
Sun et al., 2004 (79)	Intra-TM administration of galanin – antinociceptive effect	Increase in GalR1-positive neurons in the ipsilateral TM after CCI induction, but with no changes after carrageenan-induced inflammation.	Carrageenan-induced inflammation + Chronic constriction injury (CCI)	Male Wistar rats
Imbe et al., 2004 (80)	Increase in galanin-positive neurons in the arcuate nucleus of the hypothalamus in SNI animals.		Spared nerve injury (SNI)	Male Sprague–Dawley rats
Hygge-Blakeman et al., 2004 (81)	Overexpression of galanin - antinociceptive effect.		Photochemically induced sciatic nerve injury + sciatic nerve crush	Male C57/BL/6J mice (GAL-OE)
Peters et al., 2005 (82)	Tumour-bearing animals displayed increased galanin expression in ipsilateral DRG.		Sarcoma induced bone cancer	Male C3H/HeJ mice
Xiong et al., 2005 (83)	Intrathecal administration of galanin – antinociceptive effect (thermal and mechanical stimulation) in both intact rats and rats with inflammation.		Carrageenan-induced inflammation	Male Sprague-Dawley rats
Jimenez-Andrade et al., 2005 (84)	Intraplantar administration of galanin at low doses – pronociceptive effect (increases CAP-evoked nociceptive behaviours).		Capsaicin (CAP)	Male Wistar rats
Malkmus et al., 2005 (85)		GalR1 knockout mice have no differences concerning acute nociception but showed a modest tendency towards increased hyperalgesia after tissue injury and inflammation	Spinal nerve ligation (SNL)	Male C57BL/6J mice

Garry et al., 2005 (86)	Upregulation in galanin protein expression in dorsal root ganglia ipsilateral to VZV injection.		Varicella Zoster Virus (VZV) Infection	Male Wistar rats
Lu et al., 2005 (87)	The spinal content of galanin and increased gradually and reached the highest level at 24 h after colorectal distension.		Colorectal distension (CRD)	Male Sprague-Dawley rats
Holmberg et al., 2005 (88)	Overexpression of galanin leads to a high galanin expression in neuronal cell bodies in autonomic ganglia, DRG and motoneurons + high levels of galanin in nerve terminals in peripheral tissues innervated by these ganglia.		Sciatic nerve axotomy	Gal-OE mice
Jimenez-Andrade et al., 2006 (89)	Intraplantar galanin at low concentrations – pronociceptive.	Peripheral activation of GalR1 with M617 - antinociceptive effect (reduces CAP-induced inflammatory pain).<br>Peripheral activation of GalR2 with AR-M1896 - pronociceptive effect	Capsaicin (CAP)	Male Wistar rats
Shi et al., 2006 (90)		Lack of the GalR2 results in a considerable developmental loss of DRG neurons.<br>The deletion of this receptor does not influence the expression of galanin.	Spinal nerve injury	GalR2 KO mice
Brumovsky et al., 2006 (91)		Axotomy induced a strong increase in intensity and number of GalR2 positive motoneurons ipsilateral to the lesion.<br>In contrast, nerve cut or hindpaw inflammation did not alter the expression of Gal1 or Gal2 in the dorsal horn.	Sciatic nerve axotomy	Male Sprague-Dawley rats
Wilson-Gerwing & Verge, 2006 (92)	Detectable galanin mRNA is limited to a small number of DRG neurons in the intact state and is dramatically upregulated after CCI.		Chronic constriction injury (CCI) of the sciatic nerve	Male Wistar rats
Wallace et al., 2007 (93)	Increase in galanin expression in the DRG ipsilateral to virus injection.		HIV-induced painful peripheral neuropathy	Male Wistar rats

Wang et al., 2007 (94)	Peripheral nerve injury induces an expansion of the central projection of galanin-containing afferents in lamina II of the monkey spinal cord, not only by increasing galanin levels in primary afferents but also by triggering afferent branching.		Sciatic nerve axotomy	Male monkeys
Gu et al., 2007 (95)	Intra-ARC injection of galanin - antinociceptive effect (mechanical and thermal behaviour).<br>Both endogenous and exogenous galanin were involved in the regulation of nociception in the ARC of rats with peripheral nerve injury.		Sciatic nerve ligation	Male Wistar rats
Nishii et al., 2007 (96)	Galanin mRNA levels in the CP-treated group increased significantly in the ARC and the paraventricular nucleus, but not in the medial preoptic area.		Cyclophosphamide (CP)-induced cystitis (Visceral pain)	Male C57BL/6J mice
Hulse et al., 2008 (97)	Increase of galanin-expressing neurons in L3 and L4 DRG ipsilateral but not contralateral to the injury.		Partial saphenous nerve ligation injury (PSNI)	Both male and female 129/OlaHsd mice
Coronel et al., 2008 (98)	SLNC induced a gradual increase in the number of galanin-positive neurons, in direct correlation with the degree of constriction in the DRG and spinal cord.		Single ligature nerve constriction (SLNC)	Male Sprague-Dawley rats
Jung et al., 2009 (99)	High doses of galanin - antinociceptive effect (inhibited neuropathic pain behaviours).		Spared nerve injury (SNI)	Male Sprague-Dawley rats
Coronel et al., 2009 (100)	CCI induced an ipsilateral increase in the number of galanin immunoreactive neurons.		Chronic constriction injury (CCI) of the sciatic nerve	Male Sprague-Dawley rats
An et al., 2010 (101)	Subarachnoid transplantation of immortalized galanin-overexpressing astrocytes - antinociceptive effect in chronic neuropathic pain of SNI rats.		Spared nerve injury (SNI)	Male Sprague-Dawley rats
Pope et al., 2010 (102)	Overexpression of galanin – antinociceptive effect (attenuated allodynia).<br>Galanin suppression – pronociceptive effect (increase in allodynia).		Sciatic nerve axotomy	Transgenic mice (CBA/B6 strain)

Zhang et al., 2011 (103)	Galanin upregulation in DRG and sciatic nerve after nerve injury.		Chronic constriction injury (CCI) of the sciatic nerve	Male GFAP-IκBα-dn + WT mice
Hulse et al., 2011 (104)	Exogenous galanin altered the responses of mechano-nociceptive C-fibre afferents in a dose-dependent manner in both naive and nerve-injured animals, with low concentrations facilitating and high markedly inhibiting mechano-nociceptor activity.	The effects of galanin were mediated by activation of GalR2.	Partial saphenous nerve ligation injury (PSNI)	Male Wistar rats, male Gal-OE and strain matched wildtype CBA/Bl6 mice
Hulse et al., 2012 (105)	Intra-arterial infusion of galanin – antinociceptive effect (inhibits acetone and menthol responses) in the naive rodent and following models of neuropathic and inflammatory pain.<br>Overexpression of galanin after nerve injury in the DRG - antinociceptive (inhibition of cooling pain responses)	The modulatory effects of galanin on cooling are independent of GalR2 and GalR3 activation, but mediated by activation of GalR1.	Carrageenan-induced inflammation + partial sciatic nerve ligation injury (PSNI)	Male Wistar Han rats + transgenic mice
Yang et al., 2012 (106)	Upregulation of Gal in DRG and dorsal horn caused by sciatic nerve-pinch injury.<br>Exogenous Gal administration - antinociceptive role (relieving pain and shortening recovery time of pain behaviour).	Gal, GalR1 and GalR2 show great expression plasticity in DRG and dorsal horn after nerve injury and intrathecal injection of exogenous Gal.	Sciatic nerve-pinch injury	Male rats
Xu et al., 2012 (107)	Intrathecal administration of exogenous galanin – antinociceptive effect (attenuated diabetic and neuropathic pain)	(1) GalR1 expression decreased in SDH in diabetic rats; (2) GalR2 expression decreased in dorsal root ganglia and SDH in diabetic rats; (3) intrathecal administration of GalR1 agonist M617, but not GalR2 agonist AR-M1896, attenuated diabetic neuropathic pain	Streptozotocin-induced diabetes	Male Wistar rats
Amorim et al., 2014 (108)	Administration of galanin to the dorsomedial hypothalamic nucleus – pronociceptive in awake healthy and arthritic animals.<br>Increase in galanin expression in the rostral ventromedial medulla and the dorsal raphe nucleus.	The exogenous galanin pronociceptive effect was mediated by GalR1 but not GalR2.	Kaolin/Carrageenan model	Male Wistar Han rats

Su et al., 2015 (109)	Increase in galanin-expressing neurons in the DRG, both 15 and 47 days after arthritis induction.		Collagen antibody-induced arthritis (CAIA)	Male CBA mice
Yang et al., 2015 (110)	Intra-NAc injection of galanin - antinociceptive effect in rats with inflammatory pain.	Both GalR1 and GalR2 expression were upregulated in NAc of rats with inflammatory pain.	Carrageenan-induced inflammation	Male Sprague–Dawley rats
Metcalf et al., 2015 (111)		GalR2-preferring galanin analogue - antinociceptive	Carrageenan-induced inflammation + partial sciatic nerve ligation (PSNL)	Male albino CF-1 mice, Male Sprague-Dawley rats
Reinhold et al., 2015 (112)	Galanin expression was considerably augmented in damaged neurons	Increased expression of galanin receptor Gpr151 in damaged neurons	Chronic constriction injury (CCI) of the sciatic nerve	Female C57/BL6 mice
Duan et al., 2015 (113)	Intra-NAc injection of M617 and galanin - antinociceptive. Galanin-induced antinociception being stronger than M617.	The expression of GalR1 is significantly upregulated in the NAc of rats with sciatic neuropathy.<br>GalR1 mediates the antinociceptive effect of galanin in the NAc of rats with sciatic nerve ligation.	Chronic constriction injury (CCI) of the sciatic nerve	Male Sprague-Dawley rats
Boateng et al., 2015 (114)	TNT injury was associated with significant galanin immunoreactivity in both NF-200 and peripherin-immunoreactive cells, and both cell populations demonstrated dynamic and similar significant temporal profiles		Tibial nerve transection (TNT)	Male Wistar rats
Wu et al., 2016 (115)		Decreased expression of GalR1 after peripheral nerve injury	Spinal nerve ligation + Carrageenan	Male C57BL/6 mice
Xu et al., 2016 (116)	Exogenous galanin had an antinociceptive effect (alleviated the neuropathic pain) and promoted sciatic nerve regeneration more effectively in diabetic rats than after sciatic nerve pinch injury.<br>Galanin mRNA expression was dramatically upregulated after sciatic nerve pinch injury in the DRG and dorsal horn of diabetic and normal rats.	GalR1 was downregulated in the dorsal horn of diabetic rats and further decreased after sciatic nerve pinch injury.<br>GalR2 mRNA expression was downregulated in both the DRG and spinal dorsal horn of diabetic rats.	Streptozotocin-induced diabetes + Sciatic nerve pinch	Male Wistar rats

		After sciatic nerve pinch injury, GalR2 mRNA expression was upregulated in both the DRG and dorsal horn of normal and diabetic rats		
Zhang et al., 2016 (117)	Three days after orthopaedic surgery, the proportion of galanin-positive neurons was increased ipsilaterally but dropped after 2 wk.<br>After axotomy, the levels of galanin were increased after 3 days and remained up-regulated for 2 weeks, although slightly reduced.		Tibial fracture with pinning + sciatic nerve axotomy	Male C57BL/6 mice
Boltz et al., 2016 (118)		GalR3 does not mediate mechanical hyperalgesia in autoimmune arthritis	Autoimmune arthritis	Male GalR3-KO mice
Zhang et al., 2017 (119)	Intra-ACC injection of galanin - antinociceptive in rats with acute inflammation.<br>Galanin mRNA expression and galanin content increased in ACC in rats with acute inflammation.	The antinociceptive effect was attenuated by intra-ACC injection of the GalR2 antagonist M871 - involvement of GalR2 in nociceptive modulation in ACC in rats with acute inflammation.<br>Both the mRNA levels of GalR2 and the content of GalR2 in ACC increased significantly in rats with acute inflammation	Carrageenan-induced inflammation	Male Sprague-Dawley rats
Holmes et al., 2017 (120)	A marked increase in galanin mRNA levels after nerve injury.	Decrease in expression of GalR1 and GalR2 after nerve injury	Sciatic nerve axotomy	Male 129/OlaHsd mice
Coronel et al., 2017 (121)	One day after spinal cord injury, no changes in galanin mRNA expression levels.<br>In contrast to the acute phase, a significant increase in galanin mRNA levels was detected 28 days after injury.	One day after spinal cord injury, we found a significant decrease in GalR2 mRNA levels, with no changes in GalR1 mRNA expression levels.<br>The spinal cord lesion also induced a late increase in GalR1 expressA94:F94ion.	Spinal cord injury	Male Sprague–Dawley rats

		Similar to findings in the acute phase, at this time point injured animals showed reduced GalR2 mRNA levels		
(122)	Intra-ACC injection of galanin – antinociceptive effect in normal rats and rats with mononeuropathy.	GalR2 is involved in the galanin-induced antinociception in the ACC in normal rats and rats with mononeuropathy.	Chronic constriction injury (CCI) of the sciatic nerve	Male Sprague–Dawley rats
Li et al., 2017 (123)	Intra-CeA administration of galanin - antinociceptive effect in normal rats and rats with neuropathy	Upregulation of GalR1 expression in rats with neuropathy	Chronic constriction injury (CCI) of the sciatic nerve	Male Sprague–Dawley rats
Chen et al., 2018 (124)	Median nerve injury upregulated amounts of galanin-positive neurons in injured DRG.	Nerve injury upregulated amounts of GalR2-positive neurons in injured DRG.<br>Treatment with the GalR2 antagonist M871 ameliorated CCI-induced mechanical hypersensitivity, but it was exaggerated by the GalR2 agonist AR-M1896.	Median nerve chronic constriction injury (CCI)	Male Sprague–Dawley rats
Fernandez-Zafra et al., 2019 (125)	No change in expression levels of galanin		Collagen antibody-induced arthritis (CAIA)	Male and female Balb/cAnNRj, CBA (Harlan), and C57BL/6JRj
Zhang et al., 2019a (126)	Intra-NAc injection of galanin - dose-dependent antinociceptive effect (noxious thermal and mechanical stimulation) in mono-neuropathic rats and that this effect was stronger than that in intact rats.<br>Galanin expression in the NAc was upregulated after sciatic nerve ligation.	The intra-NAc injection of the non-selective GalR antagonist galantide reduced the mechanical threshold in the rats with neuropathic pain, but there was no influence of galantide in intact rats.	Chronic constriction injury (CCI) of the sciatic nerve	Male Sprague-Dawley rats

Zhang et al., 2019b (127)	M35 (galanin antagonist) in the nucleus accumbens blocked the galanin-associated antinociception of rats with mononeuropathy, but M35 alone did not affect HWLs	GalR1 activation results in the inhibition of the PKA, leading to antinociception for neuropathic pain in the NAc of rats	Chronic constriction injury (CCI) of the sciatic nerve	Male Sprague-Dawley rats
Lyu et al., 2020 (128)		Increased expression of GalR2 in DRG neurons	Spared nerve injury (SNI)	Male C57BL/6N mice
Liu et al., 2020 (129)	The transection of ION/IAN increased both galanin mRNA and peptide levels in the rat trigeminal ganglia. Galanin levels were elevated in both injured and adjacent uninjured small and medium-sized neurons after ION/IAN transection	The levels of GalR2 immunoreactivity were reduced in both injured and adjacent uninjured trigeminal ganglia neurons after ION/IAN transection, while levels of GalR1 and GalR3 remained unchanged.	infraorbital nerve (ION) and inferior alveolar nerve (IAN) transection	Male Wistar rats
Li et al., 2021 (130)		GalR2 is involved in the galanin-induced analgesic effect by activating CaMKII and PKC in the NAc of inflammatory pain rats	Carrageenan-induced inflammation	Male Sprague-Dawley rats
Dong et al., 2021 (131)		GalR2 is activated in the NAc from day 14 to day 28 after nerve injury.	Chronic constriction injury (CCI) of the sciatic nerve	Male Sprague-Dawley rats
